# Cerebellar microstructural and functional connectivity changes in patients with neuromyelitis optica spectrum disorders and their correlation with cognitive function: a female-dominated multimodal MRI study

**DOI:** 10.3389/fneur.2025.1647244

**Published:** 2025-12-09

**Authors:** Yingyu Zhang, Zhaoshi Zheng, Linfang Li, Xiaoshuang Wang, Jiebing Gu, Di Wang, Jing An, Xuemei Han

**Affiliations:** 1 First Department of Neurology, China-Japan Union Hospital of Jilin University, Changchun, Jilin Province, China; 2 The Second Affiliated Hospital of Henan University of Science and Technology, Luoyang, China

**Keywords:** neuromyelitis optica spectrum disorder, cerebellum, functional connectivity, resting-state functional magnetic resonance imaging, diffusion tensor imaging, voxel-based morphometry

## Abstract

**Background:**

The cerebellum’s role in neuromyelitis optica spectrum disorder (NMOSD) remains inadequately explored, despite its known contributions to cognition and motor function.

**Methods:**

This multimodal neuroimaging study integrated voxel-based morphometry (VBM), resting-state functional MRI (fMRI), and diffusion tensor imaging (DTI) to characterize cerebellar gray matter volume (GMV), microstructure, and functional connectivity (FC) in 29 NMOSD patients and 25 matched healthy controls. Clinical assessments included the Expanded Disability Status Scale (EDSS) and Montreal Cognitive Assessment (MoCA).

**Results:**

Patients exhibited significant cerebellar alterations, including GMV reduction in bilateral lobules VI/VIII and the vermis, decreased fractional anisotropy in Crus I, and altered FC between Crus I and occipital/frontal regions. Critically, the structural and microstructural impairments correlated with higher EDSS scores (^*^p < 0.05), while FC changes were associated with lower MoCA scores.

**Conclusion:**

These findings implicate the cerebellum in both motor disability and cognitive impairment in NMOSD, providing novel evidence for cerebellar pathology as a contributor to disease progression.

## Introduction

Neuromyelitis optica spectrum disorder (NMOSD) is an autoimmune condition primarily targeting the optic nerves and spinal cord, leading to relapsing neurological symptoms and often irreversible disability (1, 2). Beyond motor and sensory deficits, cognitive impairment is a prominent feature of NMOSD, particularly affecting memory, information processing speed, and attention (3, 4). Additionally, mood disorders such as depression and anxiety are highly prevalent in NMOSD patients, further exacerbating functional decline and reducing quality of life (5, 6). Given the significant impact of these neuropsychiatric symptoms, a comprehensive understanding of their underlying mechanisms is crucial for improving patient care.

Recent evidence suggests that the cerebellum, traditionally associated with motor coordination, plays a critical role in cognition and emotion regulation (7, 8). In NMOSD, cerebellar involvement may contribute to both cognitive deficits and mood disturbances, given its extensive connections with cortical and limbic regions (9, 10). However, the extent and nature of cerebellar alterations in NMOSD remain poorly understood.

Advances in neuroimaging techniques provide powerful tools to investigate microstructural and functional changes in the cerebellum. Resting-state functional MRI (rs-fMRI) can reveal disruptions in functional connectivity (FC) between the cerebellum and cognitive/emotional networks (11, 12).

Given the well-documented cognitive impairment in NMOSD (3, 4) and the cerebellum’s known involvement in cognitive processes (8, 13), we hypothesized that NMOSD patients would exhibit aberrations in cerebellar structure, functional connectivity, and microstructural integrity. We further hypothesized that these alterations would correlate with clinical metrics of disability and cognition, as suggested by preliminary neuroimaging evidence (14, 15). In this study, we employed 3D-T1-weighted imaging (3D-T1WI), rs-fMRI, and diffusion tensor imaging (DTI) to investigate cerebellar alterations in NMOSD. Specifically, we aimed to determine whether NMOSD patients exhibit microstructural and functional connectivity (FC) changes in the cerebellum and to examine the relationship between these cerebellar FC and microstructural abnormalities. The primary objective was to investigate how these multimodal cerebellar alterations correlate with neurological disability and cognitive impairment. Secondarily, we explored their potential relationships with mood disturbances.

## Participants and methods

### Participants

This retrospective analysis included 29 NMOSD patients admitted to the China-Japan Union Hospital of Jilin University between March 2016 and November 2022, alongside 25 demographically matched HCs. Cognitive and psychological evaluations were conducted within 1 week before and after the MRI using the Expanded Disability Status Scale (EDSS) (16), the Montreal Cognitive Assessment (MoCA) (17, 18), the Mini-Mental State Examination (MMSE) (19), the Hamilton Anxiety Rating Scale (HAMA) (20), and the Hamilton Depression Rating Scale (HAMD) (19). Clinical data, including disease duration and scores from the aforementioned assessments, were documented by two experienced neurologists. The study was conducted in line with the Declaration of Helsinki and approved by the Ethics Committee of China-Japan Union Hospital of Jilin University on June 26, 2023 (Approval Number: 2023-KYYS-137). All participants provided written informed consent.

### Inclusion and exclusion criteria

All participants were enrolled through a single, standardized screening protocol. Individuals assigned to the NMOSD group were required to satisfy the following conditions: a confirmed diagnosis of neuromyelitis optica spectrum disorder according to the 2015 International Consensus Diagnostic Criteria (2), seropositivity for anti-aquaporin-4 IgG antibodies, and first-time diagnosis; native Chinese speakers who were right-handed and younger than 65 years of age; absence of any concurrent neurological disorders; no clinical relapse within the preceding 6 weeks and no exposure to corticosteroids or disease-modifying therapies; and acquisition of all brain MRI scans on the identical scanner. Healthy controls (HC) were right-handed volunteers individually matched for age, sex, and educational level, with no evidence of systemic or neurological disease affecting the central nervous system, no abnormal neurological signs or symptoms, and no pathological findings on conventional MRI.

Exclusion criteria applied to all potential participants were the presence of intracranial lesions (e.g., cerebral infarction or space-occupying lesions) that could confound the results, a history of substance abuse or medication-induced mood disorders, severe cardiopulmonary disease, or age ≥65 years.

### Image acquisition

Two radiologists independently reviewed image quality, and one NMOSD patient and one healthy control were excluded due to severe artifacts. All participants in the HC and NMOSD groups underwent imaging using the same MRI apparatus (3.0 Tesla superconducting Magnetic Resonance Scanner, Trio Tim, Siemens Medical Systems, Erlangen, Germany). Prior to scanning, participants were familiarized with the scanner environment through a simulated scanning session with noise playback and were instructed to remain still with eyes closed but awake. Subsequently, they underwent 3D-T1WI, resting-state fMRI (rs-fMRI), and DTI. The detailed acquisition parameters for each sequence are summarized in Table 1.

**Table 1:** Magnetic resonance imaging acquisition parameters.

Parameter	3D T1-weighted imaging	Resting-state fMRI	Diffusion tensor imaging
Sequence type	MPRAGE	Gradient-echo EPI	Spin-echo EPI
Repetition time (TR)	2,300 ms	2,000 ms	3,700 ms
Echo time (TE)	2.32 ms	30 ms	95 ms
Field of view (FOV)	240 × 240 mm^2^	210 × 210 mm^2^	220 × 220 mm^2^
Acquisition matrix	256 × 256	64 × 64	110 × 110
Slice thickness	1 mm	4 mm	5.2 mm
Number of slices	192	36	25
Reconstructed voxel size	1.0 × 1.0 × 1.0 mm^3^	3.3 × 3.3 × 4.0 mm^3^	2.0 × 2.0 × 2.0 mm^3^
Other parameters		Flip angle = 90°	*b*-value = 1,000 s/mm^2^, 30 directions, 2 b0 images
Approx. scan time	~8 min	~8 min	~5 min

The total scanning duration for each participant, including setup and other sequences, was approximately 45 min.

### Data processing

Rs-fMRI, 3D-T1WI, and DTI data were processed using an integrated multimodal pipeline. The overarching strategy was to first identify structurally altered cerebellar regions through voxel-based morphometry (VBM), and then use these regions as seeds to investigate complementary functional and microstructural changes. All spatial normalization steps were performed to the MNI-152 standard space. The specific processing steps for each modality are detailed below.

### Structural MRI processing for voxel-based morphometry

T1-weighted structural images were processed using the CAT12 toolbox within SPM12. The pipeline included the following steps: (1) Images were segmented into gray matter (GM), white matter (WM), and cerebrospinal fluid (CSF); (2) The resulting GM segments were non-linearly normalized to the MNI-152 template using the high-dimensional DARTEL registration algorithm; (3) The normalized images were modulated by the Jacobian determinants derived from the spatial normalization to preserve the absolute volume information after accounting for volumetric changes introduced by warping, thus generating relative GMV maps for analysis. Finally, the modulated GMV maps were smoothed with an 8-mm full-width-at-half-maximum (FWHM) isotropic Gaussian kernel.

### VBM statistical analysis and ROI definition

Voxel-wise group comparisons of GMV between NMOSD patients and healthy controls were performed using a two-sample *t*-test in SPM12, with total intracranial volume (TIV) included as a nuisance covariate. The statistical threshold was set at a voxel-level uncorrected *p* < 0.001, with cluster-level family-wise error (FWE) correction at *p* < 0.05 to control for multiple comparisons.

Cerebellar regions of interest (ROIs) were defined directly from the results of this VBM analysis. Specifically, the significant clusters showing GMV reduction in the NMOSD group, located in the bilateral cerebellar Crus I, Crus II, and lobule VIII, were used to create the ROIs. The peak coordinates of these clusters were anatomically labeled by referring to the Automated Anatomical Labeling (AAL) atlas in MNI space. These VBM-derived ROIs, which represent structurally compromised cerebellar areas, were subsequently applied as seeds for both the resting-state functional connectivity and DTI microstructural analyses to ensure a direct and consistent link across all multimodal investigations.

### Resting-state fMRI preprocessing and functional connectivity analysis

Rs-fMRI data were preprocessed using the Data Processing Assistant for Resting-State fMRI Advanced Edition (DPARSFA v5.1). The preprocessing steps were conducted in the following sequence: (1) The first 10 volumes of each functional time-series were discarded to allow for magnetic field stabilization; (2) Slice-timing correction was applied to account for inter-slice acquisition differences; (3) Realignment was performed to correct for head motion, and subjects with maximum displacement >2.5 mm or rotation >2.5° were excluded; (4) The functional images were co-registered to the individual’s high-resolution T1-weighted image; (5) Using the deformation fields obtained from the DARTEL normalization of the structural image, the functional images were warped to the MNI-152 space; (6) Spatial smoothing was applied with a 6-mm FWHM Gaussian kernel; (7) Several nuisance covariates were regressed out from the data, including the 24-parameter model of head motion (Friston et al.), the signals from WM and CSF (CompCor method), and the global mean signal; (8) Temporal band-pass filtering (0.01–0.08 Hz) was applied to isolate low-frequency fluctuations.

For functional connectivity (FC) analysis, the mean time series was extracted from each of the predefined cerebellar ROIs. This time series was then correlated with the time series of every voxel across the whole brain. The resulting Pearson’s correlation coefficients (*r*-values) were converted to z-scores using Fisher’s *r*-to-*z* transformation to improve normality for subsequent group-level statistical analysis.

### DTI preprocessing and microstructural analysis

DTI data were preprocessed using the FMRIB Software Library (FSL, version 6.0; http://fsl.fmrib.ox.ac.uk/fsl). The preprocessing pipeline included the following steps: (1) Brain extraction: Non-brain tissue was removed using the Brain Extraction Tool (BET) (21). (2) Eddy current and motion correction: Eddy current-induced distortions and inter-volume head motion were corrected using the eddy_correct tool, which applies an affine alignment to a reference volume (22). This step did not incorporate topup for susceptibility distortion correction, as reversed phase-encoded b0 images were not acquired in this study. (3) Diffusion tensor fitting: The corrected diffusion data were then fitted to a diffusion tensor model using the dtifit tool to generate voxel-wise maps of fractional anisotropy (FA) and mean diffusivity (MD) (23).

The resulting individual FA and MD maps were subsequently normalized to the MNI-152 standard space using a two-step nonlinear registration strategy. First, a nonlinear transformation was computed from the individual’s native b0 image to their T1-weighted structural image. Subsequently, this transformation was combined with the high-dimensional DARTEL deformation field (from the structural normalization) and applied to bring the diffusion maps into MNI space.

### Clinical assessment

All participants underwent assessments with the EDSS, MoCA, MMSE, HAMA, and HAMD scales both before and 1 week after the MRI examination, with evaluations conducted by two experienced neurologists. Cognitive assessments used parallel test versions to minimize practice effects. Parallel test forms (e.g., MoCA-A/B) were employed, and stability was verified by baseline–retest correlation coefficients.

### MoCA and MMSE

The cognitive function of all subjects was evaluated using the MMSE and the MOCA (18). The assessment encompassed the following cognitive domains: visuospatial abilities, naming, memory, attention, language, abstraction, and delayed recall. Cognitive impairment was defined as MoCA <24 or MMSE < 24 (≤12 years of education) or <26 (>12 years) (24–30).

### EDSS

The EDSS was administered only to NMOSD patients, as it does not apply to healthy controls. The EDSS quantifies disability across eight functional systems, including pyramidal, cerebellar, brainstem, sensory, bowel and bladder, visual, cerebral, and other systems, by assigning a Functional System Score (FSS) to each.

### HAMA

The HAMA total score provides a comprehensive reflection of anxiety symptom severity and is utilized to evaluate anxiety severity and the efficacy of various pharmacological and psychological interventions in patients with anxiety and depressive disorders. According to data from the China Scale Collaboration Group, a total score of ≥29 indicates severe anxiety, a score of ≥21 indicates marked anxiety, a score of ≥14 indicates definite anxiety, a score of ≥7 suggests possible anxiety, and a score of <7 indicates the absence of anxiety symptoms (31–33).

### HAMD

The total score on the HAMD serves as a robust indicator of the severity of depression, with higher scores reflecting more severe depressive symptoms and lower scores indicating milder symptoms. The HAMD can be categorized into seven distinct factor structures: (1) Anxiety/Somatization, encompassing six items: psychic anxiety, somatic anxiety, gastrointestinal symptoms, general somatic symptoms, hypochondriasis, and insight; (2) Body weight, consisting of one item: weight loss; (3) Cognitive impairment, comprising six items: self-guilt, suicidal thoughts, agitation, depersonalization and derealization, paranoia, and obsessive-compulsive symptoms; (4) Diurnal variation, with one item; (5) Retardation, including four items: depression, work and interests, retardation, and sexual symptoms; (6) Sleep disturbance, incorporating three items: difficulty falling asleep, shallow sleep, and early awakening; and (7) Hopelessness, consisting of three items: feelings of helplessness, hopelessness, and worthlessness (34, 35). The HAMD score >14 was used to indicate a depressive state (33). It is important to note that the HAMA and HAMD should not be utilized in isolation; rather, a combined assessment of HAMA and HAMD scores is essential for a comprehensive evaluation of anxiety and depression severity (60).

### Statistical analysis

Demographic, clinical, and neuropsychological data were analyzed using SPSS version 29.0. Normally distributed continuous variables were expressed as mean ± standard deviation and compared using independent two-sample *t*-tests, while non-normally distributed data were expressed as median (interquartile range) and analyzed using Mann–Whitney *U* tests. Categorical variables such as the incidence of anxiety and depression were compared using chi-square tests, with statistical significance set at *p* < 0.05.

For imaging data analysis, structural MRI (VBM) comparisons of GMV between groups were performed using two-sample *t*-tests in SPM12, with results thresholded at *p* < 0.001 (uncorrected) and cluster-level family-wise error (FWE) correction (*p* < 0.05) applied using Gaussian Random Field (GRF) theory, while controlling for total intracranial volume (TIV) as a covariate. Functional connectivity (rs-fMRI) differences were similarly analyzed using two-sample t-tests in SPM12 with voxel-level significance set at *p* < 0.001 and cluster-level FWE correction (*p* < 0.05), incorporating head motion parameters as nuisance covariates. For DTI analysis, group comparisons of FA/MD values were conducted using two-sample *t*-tests in SPM12 with GRF correction (voxel-level *p* < 0.001, cluster-level FWE *p* < 0.05), adjusting for age and sex as covariates across all imaging analyses.

Correlation analyses between imaging measures (FC, FA, MD, GMV) and clinical scores (EDSS, MoCA, MMSE, HAMA, HAMD) were performed in SPSS using Pearson correlation for normally distributed data and Spearman correlation for non-normally distributed data, with a significance threshold of *p* < 0.05 after false discovery rate (FDR) correction for multiple comparisons. All imaging statistical models incorporated appropriate multiple comparison corrections as specified to ensure robust statistical inference.

## Results

### General characteristics and clinical scores of participants

The study included 29 patients diagnosed with NMOSD and 25 healthy volunteers matched for sex, age, and education. Within the NMOSD cohort, there was 1 male and 28 females, with ages ranging from 20 to 65 years and a mean age of 45.03 ± 12.59 years. Their educational attainment ranged from 6 to 16 years, with a mean of 10.90 ± 2.85 years. The HC group comprised 2 males and 23 females, aged 24 to 63 years, with a mean age of 46.08 ± 9.92 years, and educational attainment ranging from 6 to 16 years, with a mean of 11.08 ± 3.14 years. Statistical analysis revealed no significant differences between the two groups concerning age, sex, or educational level (*p* > 0.05). Table 1 presents the scores for the EDSS, MMSE and MoCA, HAMA, and HAMD for both groups. The incidence of cognitive impairment in the NMOSD group was 82.8%. Compared with the HC group (*p* < 0.05), patients with NMOSD had significantly reduced cognitive function. Baseline–retest correlations showed good reliability (MoCA *r* = 0.88, MMSE *r* = 0.82, both *p* < 0.001). The difference in the prevalence of depression and anxiety between the two groups (*p* < 0.05) was statistically significant (Table 2).

**Table 2:** Comparison of demographic and clinical characteristics between the NMOSD and HC groups.

Variables	NMOSD group (*n* = 29)	HC group (*n* = 25)	*t*/*χ*^2^	*p*-value
Sex (male/female, *n*)	1/28	2/23	2.87	0.09
Age (year)	45.03 ± 12.59	46.08 ± 9.92	−0.27	0.79
Education level (year)	10.90 ± 2.85	11.08 ± 3.14	−0.47	0.64
MoCA score	21.16 ± 4.17	25.08 ± 2.58	−5.84	<0.001
MMSE score	25.24 ± 4.99	26.92 ± 2.66	−2.55	0.016
HAMD score	6.00 (4.50, 9.00)	3.00 (2.00, 5.00)	−2.02	0.043
HAMA score	7.00 (4.00,9.00)	4.00 (3.00, 6.00)	−2.30	0.021
EDSS score	3.50 (2.50, 4.25)	—	—	—
Cognitive impairment	24	4	15.30	<0.001
Anxious state	3	3	0.001	1.00
Depression state	2	2	0.10	0.60

Normally distributed data were expressed as mean ± standard deviation (SD) and analyzed using the independent sample *t*-test, while non-normally distributed data were expressed as medians (with the lower and upper quartiles), and analyzed using non-parametric test on two independent samples. *p* < 0.05 indicated that the differences were statistically significant. NMOSD, neuromyelitis optica spectrum disorder; HC, healthy control; EDSS, Expanded Disability Status Scale; MMSE, Mini-Mental State Examination; MoCA, Montreal Cognitive Assessment; HAMA, Hamilton Anxiety Rating Scale, HAMD, Hamilton Depression Rating Scale.

### VBM analysis results

**Figure 1:**
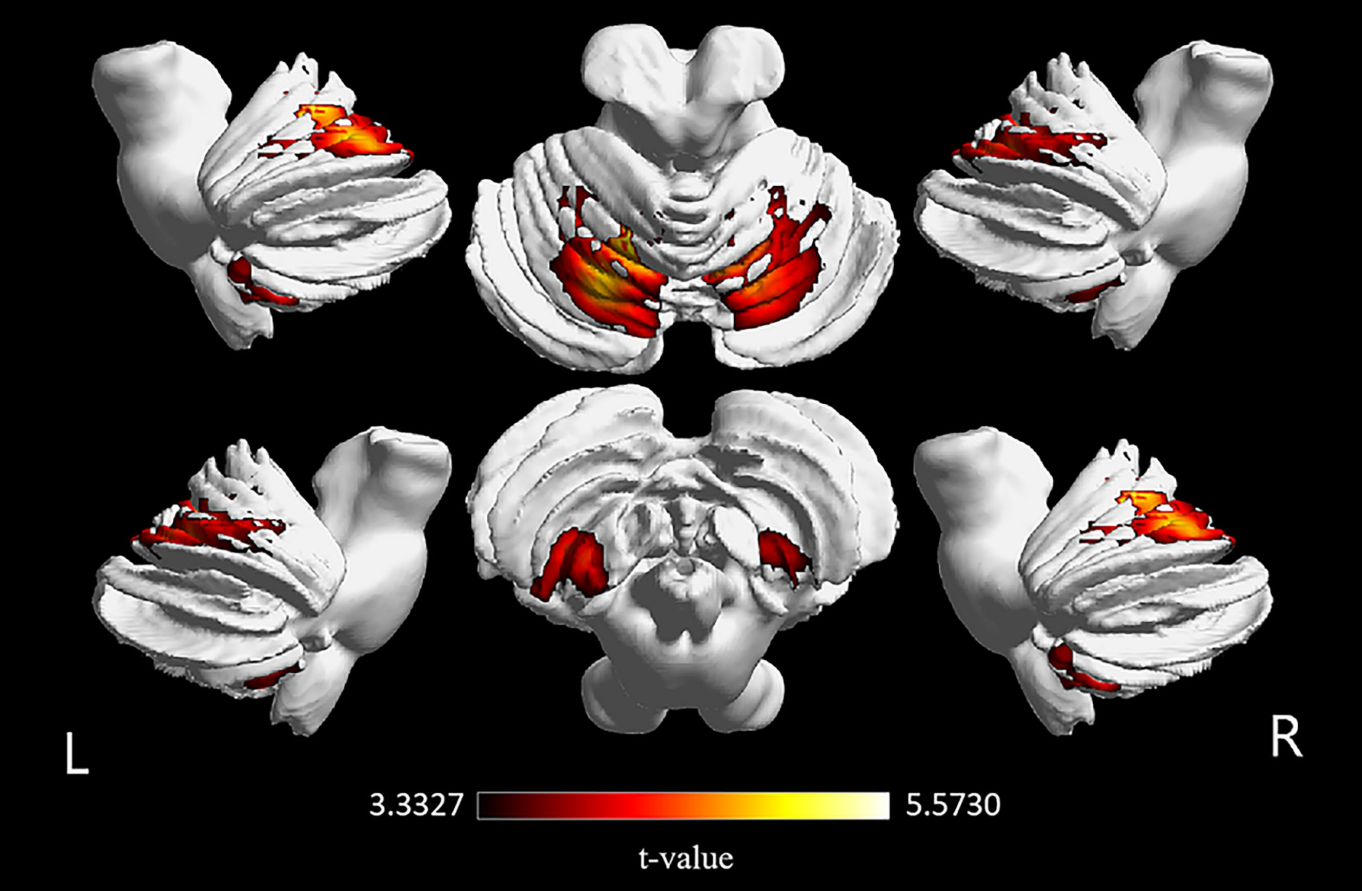
Comparison of cerebellar GMV between the NMOSD and HC groups. The NMOSD group showed significantly reduced cerebellar GMV in the bilateral cerebellar lobule VIII, left cerebellar lobules IX, cerebellar vermis VI, left cerebellar Crus I and Crus II, and bilateral cerebellar lobule VI compared with the HC group (*p* < 0.001, FWE corrected, cluster size >1,058 voxels). NMOSD, neuromyelitis optica spectrum disorder; HC, healthy control; GMV, gray matter volume.

VBM analysis identified a significant reduction in cerebellar GMV in the NMOSD group compared to the HC group. Specifically, reductions were observed in the right cerebellar lobule VIII, left cerebellar lobules VIII and IX, cerebellar vermis VI, left cerebellar Crus I and Crus II, and bilateral cerebellar lobule VI [*p* < 0.001, family-wise error (FWE) corrected, cluster size >1,058 voxels]. Differential clustering coordinates and statistics value extracted from these cerebellar sub-regions, including bilateral cerebellar Crus I, Crus II, and lobule VIII, are presented in Table 3 and Figure 1.

**Table 3:** Cerebellar regions with significant cerebellar GMV differences between the NMOSD and HC groups.

Brain regions (AAL)	Cluster size (mm^3^)	MNI coordinates	*T* value	Brain regions (AAL)	Cluster size (mm^3^)
		*X*	*Y*	*Z*	
Right cerebellar lobule VIII	1,688	27	−39	−55.5	4.970
Left cerebellar lobules VIII and IX	1,058	−36	−34.5	−52.5	4.784
Bilateral cerebellar lobule VI, left cerebellar Crus I and Crus II, cerebellar vermis VI	6,494	−10.5	−64.5	−27	5.573

MNI coordinates mean the peak coordinates, which indicate cerebellar brain regions with significantly reduced cerebellar GMV in patients with NMOSD compared with HCs. The significance level was set at *p* < 0.01 corrected by FWE, with a cluster size >1,058 voxels. NMOSD, neuromyelitis optica spectrum disorder; HC, healthy control; GMV, gray matter volume; AAL, automated anatomical labeling template(Only used to locate the cluster center); MNI: Montreal Neurological Institute.

### Differences in cerebellar FC between the NMOSD and HC groups

**Figure 2:**
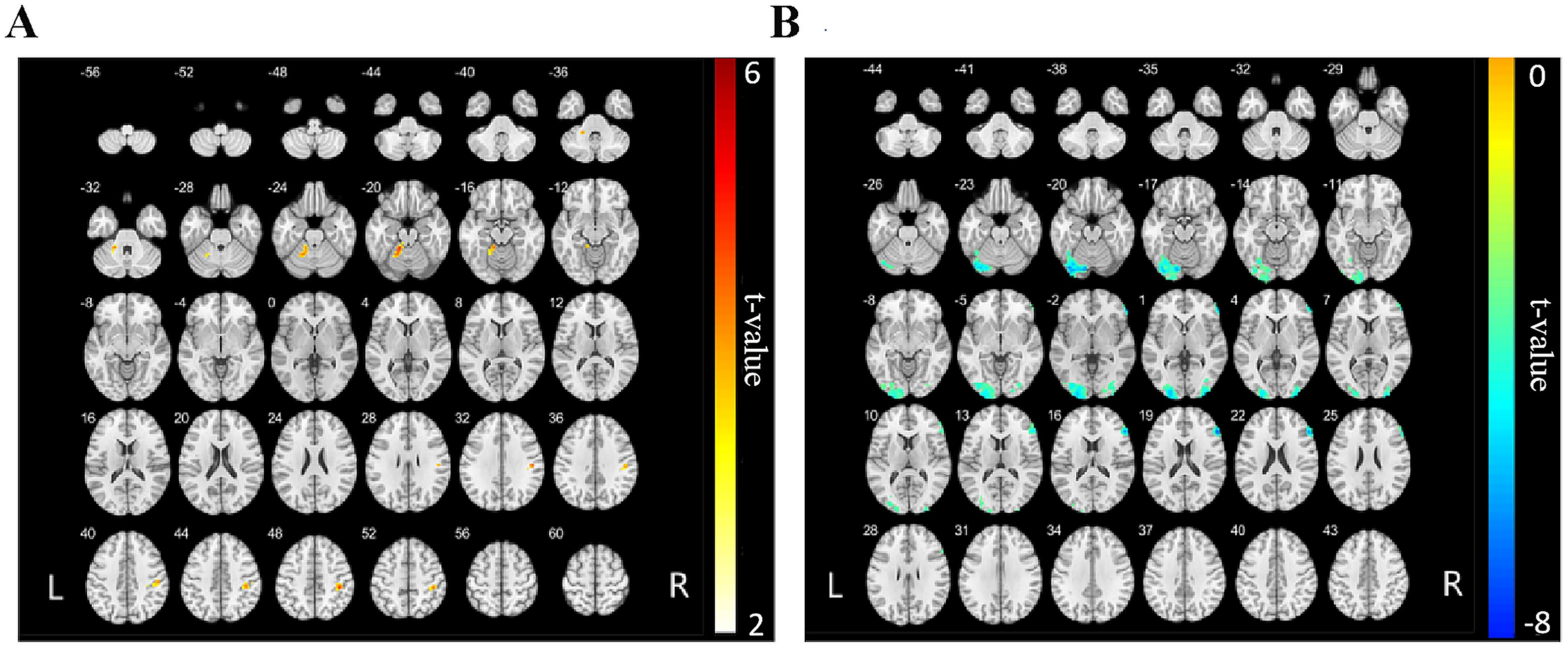
FC map of the bilateral cerebellar Crus I and whole-brain regions between the NMOSD and HC groups. **(A)** The red areas represent brain regions exhibiting increased FC with the left cerebellar Crus I. The deeper the color, the stronger the color, including left cerebellar lobules IV, V, VI, left fusiform gyrus, right postcentral gyrus, and right supramarginal gyrus (*p* < 0.001, FWE corrected, cluster size >77 voxels). **(B)** The blue areas represent brain regions of reduced FC to the right cerebellar Crus I, including left middle occipital gyrus, left cerebellar Crus I, left inferior occipital gyrus, right middle occipital gyrus, right inferior occipital gyrus, right triangular part of the inferior frontal gyrus, and right middle frontal gyrus (*p* < 0.001, FWE corrected, cluster size >112 voxels). NMOSD, neuromyelitis optica spectrum disorder; HC, healthy control; FC, functional connectivity.

Furthermore, relative to the HC group, NMOSD patients exhibited increased FC between the left cerebellar Crus I and the left cerebellar lobules IV, V, VI, left fusiform gyrus, right postcentral gyrus, and right supramarginal gyrus (*p* < 0.001, FWE corrected, cluster size >77 voxels, Table 4 and Figure 2). Conversely, a decrease in FC was noted between the right cerebellar Crus I and several regions, including the left middle occipital gyrus, left cerebellar Crus I, left inferior occipital gyrus, right middle occipital gyrus, right inferior occipital gyrus, right triangular part of the inferior frontal gyrus, and right middle frontal gyrus (*p* < 0.001, FWE corrected, cluster size > 112 voxels, Table 4 and Figure 2). FC changes between the remaining cerebellar regions and the whole-brain regions were not observed.

**Table 4:** Brain regions showing significantly altered FC with the bilateral cerebellar Crus I in the NMOSD group compared with the HC group.

Description	Brain region (AAL template)	Cluster size (mm^3^)	MNI coordinates			*T* value
			*X*	*Y*	*Z*	
Left cerebellar Crus I (NMOSD > HCs)	Left cerebellar lobules IV, V, VI, left fusiform gyrus	105	−15	−45	−21	4.915
	Right postcentral gyrus, right supramarginal gyrus	77	51	−21	30	4.760
Right cerebellar Crus I (NMOSD < HCs)	Left middle occipital gyrus, left cerebellar Crus I, left inferior occipital gyrus	663	−39	−81	−21	−6.719
	Right middle occipital gyrus, right inferior occipital gyrus	112	33	−99	0	−5.293
	Right triangular part of the inferior frontal gyrus, and right middle frontal gyrus	129	54	30	18	−7.049

NMOSD, neuromyelitis optica spectrum disorder; HC, healthy control; FC, functional connectivity; MNI: Montreal Neurological Institute; AAL, automated anatomical labeling template (only used to locate the cluster center). MNI coordinates mean the peak coordinates. Brain regions exhibiting significantly increased FC with the left cerebellar Crus I in the NMOSD group compared with the HC group (*p* < 0.001, FWE corrected, cluster size >77 voxels, *T* value >0); brain regions exhibiting significantly decreased FC with the right cerebellar Crus I in the NMOSD group compared with the HC group (*p* < 0.001, FWE corrected, cluster size >112 voxels, *T* value >0).

### Difference in FA and MD values between the NMOSD and HC groups

**Figure 3:**
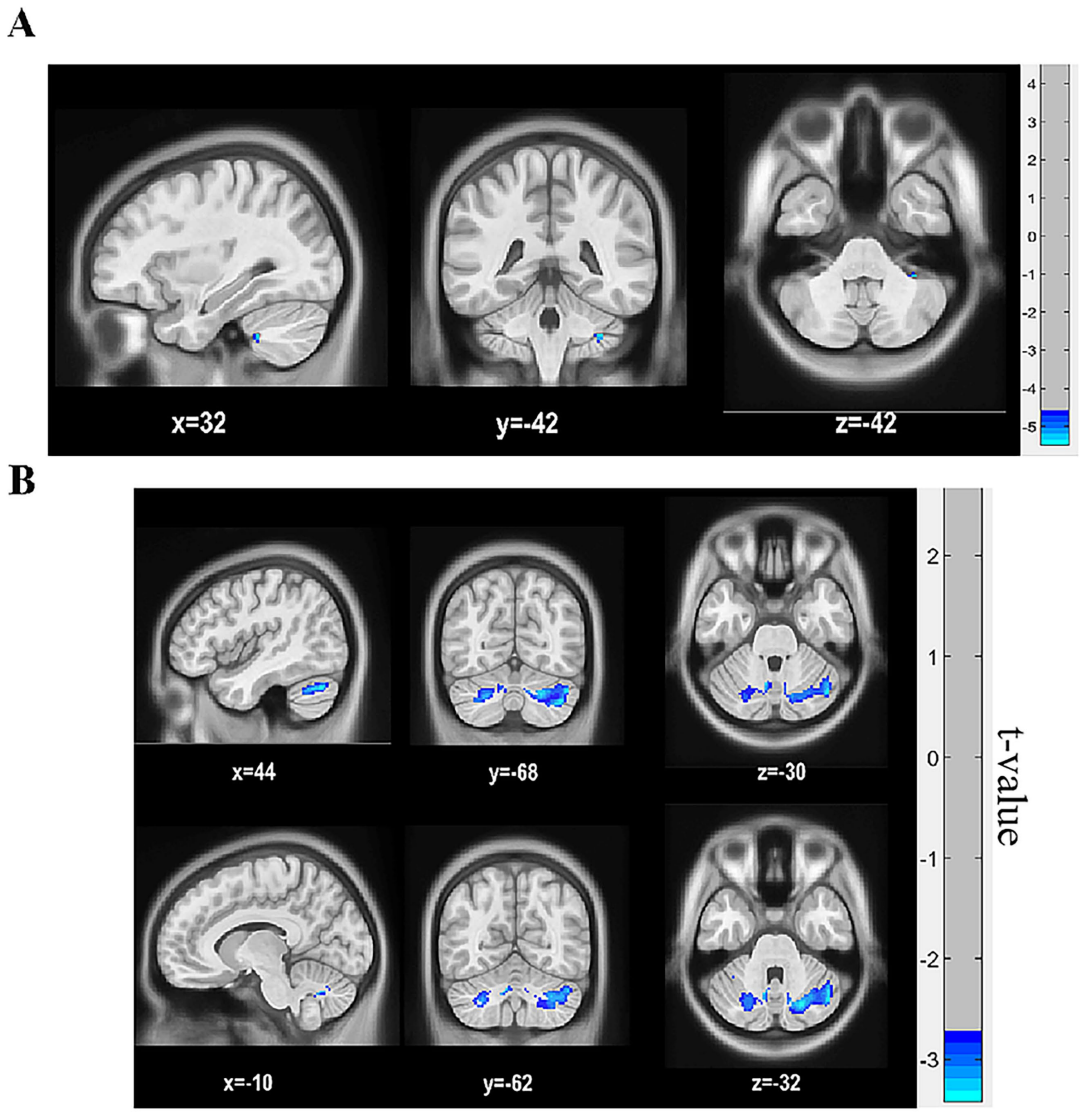
Differences in FA and MD values in cerebellar sub-regions between the NMOSD and HC groups. **(A)** Differences in FA maps between the two groups. Blue color shows reduced FA values in the right cerebellar Crus I in patients with NMOSD compared to HCs (*p* < 0.05, FWE corrected), the deeper the color, the stronger the color. **(B)** Differences in MD maps between the two groups. Blue color shows reduced MD values in bilateral cerebellar Crus I and bilateral cerebellar lobule VIII in patients with NMOSD compared to HCs (*p* < 0.05, GRF corrected, cluster size >365 voxels). NMOSD, neuromyelitis optica spectrum disorder; HC, healthy control; MD, mean diffusivity.

In comparison to th HC group, patients with NMOSD demonstrated significantly reduced FA values in the right cerebellar Crus I (*p* < 0.05, family-wise error [FWE] corrected) and significantly reduced MD values in both the bilateral cerebellar Crus I and bilateral cerebellar lobule VIII [*p* < 0.05, Gaussian random field (GRF) corrected, cluster size > 365 voxels, as shown in Tables 5, 6 and Figure 3]. No significant differences in FA and MD values were observed in the remaining cerebellar regions between the two groups.

**Table 5:** Cerebellar regions showing significantly reduced FA in the NMOSD group compared with the HC group.

Brain region (AAL template)	Cluster size (mm^3^)	MNI coordinates			*T* value
		*X*	*Y*	*Z*	
Right cerebellar Crus I	8	32	−42	−42	−5.39

NMOSD, neuromyelitis optica spectrum disorder; HC, healthy control; FA, functional anisotropy, MNI: Montreal Neurological Institute; AAL, automated anatomical labeling template (only used to locate the cluster center). MNI coordinates mean the peak coordinates. The significance level was set at *p* < 0.05 corrected by FWE.

**Table 6:** Cerebellar regions showing significantly reduced MD in the NMOSD group compared with the HC group.

Brain region (AAL template)	Cluster size (mm^3^)	MNI coordinates			*T* value
		*X*	*Y*	*Z*	
Right cerebellar Crus I/right cerebellar lobule VIII	989	44	−68	−30	−3.42
Left cerebellar Crus I/left cerebellar lobule VIII	420	−10	−62	−32	−3.30

NMOSD, neuromyelitis optica spectrum disorder; HC, healthy control; MD, mean diffusivity; MNI, Montreal Neurological Institute; AAL, automated anatomical labeling template (only used to locate the cluster center). MNI coordinates mean the peak coordinates. The significance level was set at *p* < 0.05 corrected by GRF, with a cluster size >365 voxels.

### Correlation between cerebellar structural and functional changes, as well as their associations with neurological disability and cognitive function in patients with NMOSD

FC, FA, MD, and GMV values from brain regions displaying significant alterations in the bilateral cerebellar Crus I in the NMOSD group, as compared to the HC group, were extracted for further analysis. Pearson correlation analyses were conducted to investigate the relationships between FC values and FA, MD, and GMV values within the NMOSD group. Subsequently, correlations between these values and the EDSS, MoCA, HAMD, and HAMA scores were evaluated.

**Figure 4:**
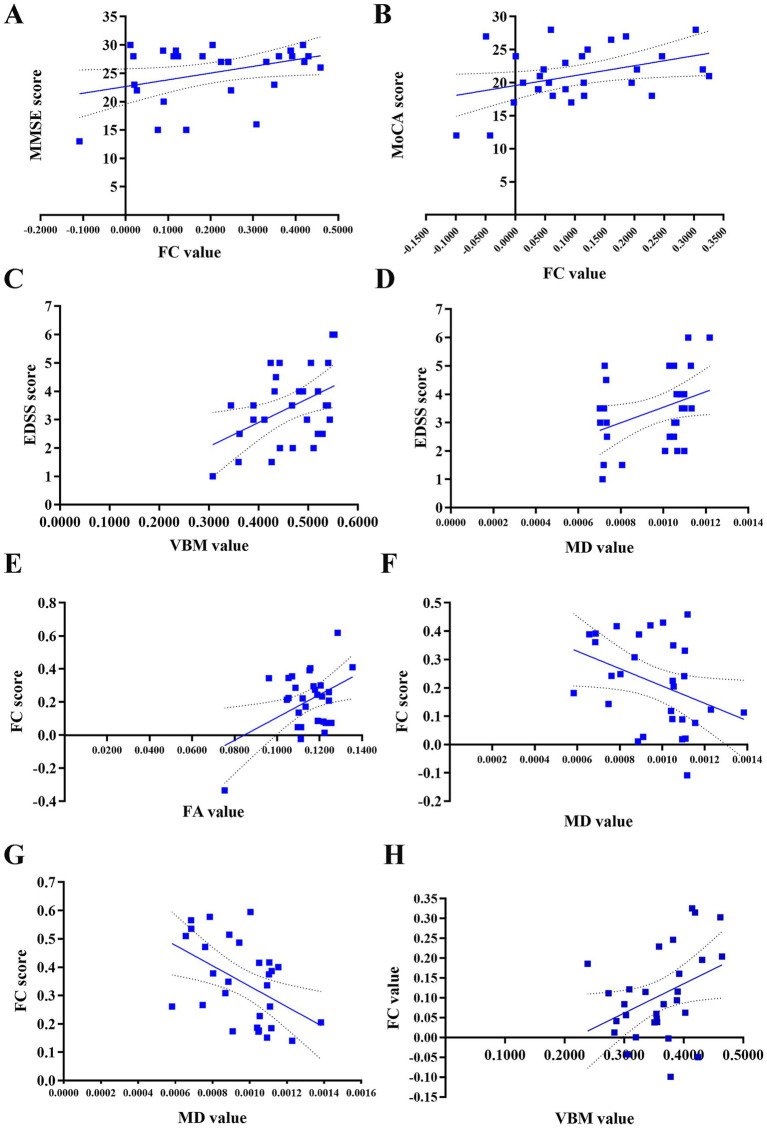
Correlation between FC values and FA, MD, GMV values, and their associations with clinical scores in patients with NMOSD. **(A)** Positive correlation of the FC value between the right cerebellar Crus I and the left middle occipital gyrus with the MMSE score (*r* = 0.372, *p* = 0.047). **(B)** Positive correlation of the FC value between the right cerebellar Crus I and the right triangular part of inferior frontal gyrus with the MoCA score (*r* = 0.394, *p* = 0.034). **(C)** Positive correlation between the GMV value in the bilateral cerebellar lobule VI and the EDSS score (*r* = 0.392, *p* = 0.036). **(D)** Positive correlation between the MD value in the right cerebellar Crus I and the EDSS score (*r* = 0.398, *p* = 0.033). **(E)** Positive correlation of the FC value between the left cerebellar Crus I and the right postcentral gyrus with the FA value (*r* = 0.439, *p* = 0.017). **(F)** Negative correlation of the FC value between the right cerebellar Crus I and the right middle occipital gyrus with the MD value (*r* = −0.374, *p* = 0.046). **(G)** Negative correlation of the FC value between the right cerebellar Crus I and the left middle occipital gyrus with the MD value (*r* = −0.480, *p* = 0.008). **(H)** Negative correlation of GMV value in the right cerebellar Crus I with the FC value between the right cerebellar Crus I and right triangular part of the inferior frontal gyrus (*r* = −0.383, *p* = 0.04). NMOSD, neuromyelitis optica spectrum disorder, FC, functional connectivity; GMV, gray matter volume; FA, fractional anisotropy, MD, mean diffusivity; EDSS, Expanded Disability Status Scale; MMSE, Mini-Mental State Examination; MoCA, Montreal Cognitive Assessment.

The study found several correlations in the NMOSD group (Figure 4): (1) Positive correlation between FC value (right cerebellar Crus I-left middle occipital gyrus) and MMSE score (*r* = 0.372, *p* = 0.047); (2) Positive correlation between FC value (right cerebellar Crus I-right triangular part of inferior frontal gyrus) and MoCA score (*r* = 0.394, *p* = 0.034); (3) Positive correlations of GMV in bilateral cerebellar lobule VI and MD in right cerebellar Crus I with EDSS score (*r* = 0.392, *p* = 0.036; r = 0.398, *p* = 0.033); (4) Positive correlation between FC value (left cerebellar Crus I-right postcentral gyrus) and FA value (*r* = 0.439, *p* = 0.017); (5) Negative correlations between FC values (right cerebellar Crus I-right/left middle occipital gyrus) and MD value in right cerebellar Crus I (*r* = −0.374, *p* = 0.046; *r* = −0.480, *p* = 0.008); (6) Negative correlation between GMV in right cerebellar Crus I and FC value (right cerebellar Crus I-right triangular part of the inferior frontal gyrus) (*r* = −0.383, *p* = 0.04). No statistically significant correlation was found between the GMV or FC values in other brain regions and HAMD/HAMA scores (*p* > 0.05, Supplementary Figure 1).

## Discussion

In recent years, cognitive dysfunction and mood disorders in patients with NMOSD have gained increasing attention. Research indicates that the cerebellum, traditionally associated with motor coordination, plays a critical role in cognitive processing via connections between Crus I/II and the executive control network (36, 37). Rs-fMRI studies have revealed reorganization within working memory networks and alterations in FC of the default mode network in NMOSD patients. Reduced FC between cerebellar and visual networks may reflect underlying pathological damage, while enhanced FC in certain networks could represent compensatory mechanisms (14, 15). Furthermore, advanced neuroimaging techniques such as DTI enable the detection of microstructural alterations not visible on conventional MRI, providing novel insights into demyelination and axonal damage in NMOSD. This study aims to investigate the relationship between cerebellar structural and functional alterations and neurological disability and cognitive impairment in NMOSD patients.

In this study, we utilized VBM, fMRI, and DTI to examine alterations in cerebellar GMV, microstructure, and FC in patients with NMOSD. The results demonstrated a reduction in FC between the right cerebellar Crus I and the frontal and occipital lobes, suggesting potential disruption and reorganization of the cerebellar network in NMOSD patients. A study by Bigaut et al. (38) on resting-state FC and the topological properties of the cerebellar network in NMOSD patients revealed decreased FC in the visual and sensorimotor networks, with abnormal cerebellar network metrics correlating with disability progression. The findings of this study indicated reduced FC between the right cerebellar Crus I and the bilateral middle occipital gyrus, as well as the bilateral inferior occipital gyrus. This reduction may be linked to visual impairment resulting from optic neuritis, cerebellar-cerebral disconnection, and subsequent deficits in visual memory processing (39). Dysfunction within the visual cortex can lead to impaired visual memory and processing, thereby contributing to broader cognitive deficits in patients with NMOSD (40, 41). The findings of the current study align with previous research indicating reduced FC in primary visual areas, suggesting that the integration of the visual network may be compromised in NMOSD patients (41). Various factors may influence FC in the visual cortex of NMOSD patients, including gray matter atrophy, structural and functional alterations in connected regions, the dependence of subregions on visual input, and functional reorganization. Additionally, isolated optic nerve involvement is more prevalent among NMOSD patients. A previous study suggests that astrocyte damage and high expression of AQP4 antibodies may play an important role in the occurrence of optic neuritis (42).

In this study, we observed that patients with NMOSD demonstrated increased FC between the left cerebellar Crus I and the right postcentral gyrus compared to the HC group. The postcentral gyrus, which encompasses the primary somatosensory cortex, is responsible for receiving sensory information, including both deep and superficial sensations, from the contralateral side of the body. It plays a crucial role in the integration of somatosensory information, attention, and speech perception. Allen et al. (43) were the first to analyze whole-brain regions exhibiting signal fluctuations associated with low-frequency fluctuations in the MR signal of the dentate nucleus from a functional perspective. Their findings revealed functional coherence between the dentate nucleus and the parietal and prefrontal cortices in the human brain, indicating the existence of cerebellar-parietal and cerebellar-prefrontal FC. This conclusion was further supported by O’Reilly et al. (44), who provided anatomical, clinical, and neuroimaging evidence suggesting regional differences within the cerebellum. Connections between the cerebellum and cerebrum primarily occur via the cerebellar-basal ganglia-thalamo-cerebral circuit and the cerebellar-ponto-cerebral circuit (45). It is hypothesized that neuronal damage within the brain may lead to a compensatory increase in FC between the cerebellum and cerebrum (46–48).

However, the findings of this study demonstrated a reduction in FC between the right cerebellar Crus I and the right triangular part of the inferior frontal gyrus. This alteration was associated with cognitive function in patients with NMOSD, suggesting functional disconnections between the cerebellum and cerebrum in these patients, which may impact their cognitive abilities. The frontal lobe is involved in a variety of functions, including information integration, execution, and control. The inferior frontal gyrus serves as a critical cortical node in circuits related to cognitive control, and is commonly linked to emotion regulation and cognitive impairment. The triangular portion of the inferior frontal gyrus is a critical brain region situated within the medial prefrontal lobe. Aberrant FC between the inferior frontal gyrus and other brain regions has been observed in individuals with bipolar disorder (49). A meta-analysis has demonstrated the spatio-functional diversity of the left inferior frontal gyrus in the processing of language and working memory (50). The frontal lobe is recognized as a vital region for cognitive function. Studies have identified reduced FC in the frontal lobe and a correlation between frontal lobe activity and cognitive impairment in patients with NMOSD (51), further underscoring the significance of the frontal lobe in cognitive processes.

In this study, we observed that patients with NMOSD exhibited decreased FA values in the right cerebellar Crus I and reduced MD values in both the bilateral cerebellar Crus I and bilateral cerebellar lobule VIII, in comparison to the HC group. Correlation analysis demonstrated a positive association between the MD value of the right cerebellar Crus I and the EDSS score. The FA value serves as an imaging marker for the integrity of the myelin sheath, with reductions in FA indicating compromised integrity of fiber bundles and myelin sheaths. The MD value reflects the diffusion capacity of water molecules within tissue, which is directly proportional to this capacity. Prior research has indicated that individuals with neuromyelitis optica exhibit significantly lower average FA, higher average MD, and substantial deficits in learning and memory abilities relative to HCs (6). In contrast, Pichiecchio et al. (52) reported that while the FA value of normal-appearing GM was lower in NMOSD patients compared to the HC group, no significant difference in MD values was observed between the two groups. Our observation of concomitantly reduced FA and MD in NMOSD deviates from several prior reports. Our study revealed a distinct pattern of concomitant reduction in both FA and MD within the cerebellar Crus I and lobule VIII of NMOSD patients, a finding that contrasts with the more common pattern of decreased FA and increased MD observed in classic white matter demyelination or degeneration (53). To reconcile this, we propose a primary hypothesis centered on acute astrocytic damage and consequent cytotoxic edema as the underlying mechanism. Given the specific targeting of AQP4 on astrocytic end-feet by IgG antibodies in NMOSD (54), the initial pathological insult is likely a combination of inflammatory edema and direct astrocytic injury. This can lead to a transient reduction in the extracellular space, restricting water diffusion and thereby lowering MD, while concomitant disruption of the neuropil and axonal integrity reduces FA. This pattern of restricted diffusion is analogous to that seen in the hyperacute phase of ischemic stroke, where cytotoxic edema is the dominant process (55–57). The significant positive correlation we observed between MD in the right Crus I and the EDSS score (*r* = 0.398, *p* = 0.033) further supports this hypothesis. We interpret this to mean that within the NMOSD patient group, those with more severe clinical disability (higher EDSS) had a greater magnitude of this acute, diffusion-restricting pathology at the time of scanning. In this context, a lower MD value may serve as an *in vivo* marker of more intense acute inflammatory-cytotoxic activity, which directly translates to worse neurological function.

This primary mechanism of AQP4-mediated astrocytopathy offers a more parsimonious explanation than classical demyelination for the changes in our VBM-defined cerebellar gray matter ROIs. The cerebellar cortex, with its densely packed granular cell layer and complex synaptic architecture, may exhibit diffusion metrics that are particularly sensitive to astrocytic dysfunction and cellular swelling (58, 59). The altered functional connectivity observed between the cerebellum and cognitive/visual regions may represent both the direct disruption of cerebro-cerebellar circuits by this microstructural damage and subsequent attempts at network-level reorganization. While we cannot rule out contributions from other processes, such as microstructural reorganization or gliosis, the acute cytotoxic model most coherently unifies our DTI findings, their clinical correlation, and the known pathophysiology of NMOSD.

This study acknowledges several important limitations that merit consideration. First, the exclusive focus on AQP4-IgG-seropositive NMOSD patients limits generalizability to seronegative individuals or those with MOG antibody–associated disease (MOGAD), who may exhibit distinct patterns of cerebellar involvement. Additionally, although the marked female predominance in our cohort reflects the epidemiology of NMOSD, the findings cannot be extrapolated to male patients; future studies with gender-balanced cohorts are therefore warranted. Methodologically, the cross-sectional design combined with a moderate sample size (*n* = 54) constrains statistical power for subgroup analyses and precludes causal inferences regarding cerebellar degeneration trajectories. Critically, the absence of longitudinal data leaves cerebellar microstructural dynamics during disease progression—particularly across relapse-remission transitions-uncharacterized. Furthermore, the assessment of cognitive function relied on the MoCA and MMSE, which are global screening instruments. While effective for identifying overall cognitive impairment, they lack the sensitivity to finely characterize specific cerebellar-mediated cognitive domains such as executive function, planning, and visuospatial working memory. The inclusion of more domain-specific tests (e.g., Trail Making Test, Stroop, Digit Span) in future studies would provide a more nuanced understanding of the cognitive profile in NMOSD. Other technical limitations include possible partial volume effects in cerebellar subregions due to DTI spatial resolution constraints, alongside the lack of advanced myelin-specific imaging techniques like quantitative magnetization transfer (qMT). Future multi-center studies featuring extended follow-up (≥3 years) should integrate serial neurophysiological assessments (e.g., visual evoked potentials for visual pathway integrity), standardized longitudinal cognitive testing incorporating domain-specific measures, and serum/CSF biomarker profiling to establish temporal trajectories of cerebellar degeneration and elucidate its causal role in disability progression.

In conclusion, this study utilized a multimodal approach, integrating structural and functional MRI, to examine the microstructural and FC changes in the cerebellum of patients with NMOSD from multiple perspectives. The findings indicate a significant interaction and close association between cerebellar structural and functional alterations, which are correlated with neurological disability and cognitive function in patients with NMOSD.

## Data Availability

The original contributions presented in the study are included in the article/Supplementary material, further inquiries can be directed to the corresponding author.

## References

[1] Wingerchuk DM Lennon VA Lucchinetti CF Pittock SJ Weinshenker BG. The spectrum of neuromyelitis optica. Lancet Neurol. (2007) 6:805–15. doi: 10.1016/S1474-4422(07)70216-8, 17706564

[2] Wingerchuk DM Banwell B Bennett JL Cabre P Carroll W Chitnis T . International consensus diagnostic criteria for neuromyelitis optica spectrum disorders. Neurology. (2015) 85:177–89. doi: 10.1212/WNL.0000000000001729, 26092914 PMC4515040

[3] Blanc F Zéphir H Lebrun C Labauge P Castelnovo G Fleury M . Cognitive functions in neuromyelitis optica. Arch Neurol. (2008) 65:84–8. doi: 10.1001/archneurol.2007.16, 18195143

[4] Meng H Xu J Pan C Cheng J Hu Y Hong Y . Cognitive dysfunction in adult patients with neuromyelitis optica: a systematic review and meta-analysis. J Neurol. (2017) 264:1549–58. doi: 10.1007/s00415-016-8345-3, 27909800

[5] Akaishi T Nakashima I Misu T Fujihara K Aoki M. Depressive state and chronic fatigue in multiple sclerosis and neuromyelitis optica. J Neuroimmunol. (2015) 283:70–3. doi: 10.1016/j.jneuroim.2015.05.007, 26004160

[6] He D Chen X Zhao D Zhou H. Cognitive function, depression, fatigue, and activities of daily living in patients with neuromyelitis optica after acute relapse. Int J Neurosci. (2011) 121:677–83. doi: 10.3109/00207454.2011.608456, 21797812

[7] Schmahmann JD Ko R MacMore J. The human basis pontis: motor syndromes and topographic organization. Brain. (2004) 127:1269–91. doi: 10.1093/brain/awh138, 15128614

[8] Stoodley CJ Schmahmann JD. Functional topography in the human cerebellum: a meta-analysis of neuroimaging studies. NeuroImage. (2009) 44:489–501. doi: 10.1016/j.neuroimage.2008.08.039, 18835452

[9] Bostan AC Strick PL. The basal ganglia and the cerebellum: nodes in an integrated network. Nat Rev Neurosci. (2018) 19:338–50. doi: 10.1038/s41583-018-0002-7, 29643480 PMC6503669

[10] Li Y Yang L Li L Xie Y Fang P. The resting-state cerebro-cerebellar function connectivity and associations with verbal working memory performance. Behav Brain Res. (2022) 417:113586. doi: 10.1016/j.bbr.2021.113586, 34536430

[11] Biswal B Yetkin FZ Haughton VM Hyde JS. Functional connectivity in the motor cortex of resting human brain using echo-planar MRI. Magn Reson Med. (1995) 34:537–41. doi: 10.1002/mrm.1910340409, 8524021

[12] Kang D In MH Jo HJ Halverson MA Meyer NK Ahmed Z . Improved resting-state functional MRI using multi-echo echo-planar imaging on a compact 3T MRI scanner with high-performance gradients. Sensors. (2023) 23:4329. doi: 10.3390/s23094329, 37177534 PMC10181561

[13] Schmahmann JD Sherman JC. The cerebellar cognitive affective syndrome. Brain. (1998) 121:561–79. doi: 10.1093/brain/121.4.561 9577385

[14] Savoldi F Rocca MA Valsasina P Riccitelli GC Mesaros S Drulovic J . Functional brain connectivity abnormalities and cognitive deficits in neuromyelitis optica spectrum disorder. Mult Scler. (2020) 26:795–805. doi: 10.1177/1352458519845109, 31079538

[15] Han Y Liu Y Zeng C Luo Q Xiong H Zhang X . Functional connectivity alterations in neuromyelitis optica spectrum disorder: correlation with disease duration and cognitive impairment. Clin Neuroradiol. (2020) 30:559–68. doi: 10.1007/s00062-019-00802-3, 31578601

[16] Wingerchuk DM Lennon VA Pittock SJ Lucchinetti CF Weinshenker BG. Revised diagnostic criteria for neuromyelitis optica. Neurology. (2006) 66:1485–9. doi: 10.1212/01.wnl.0000216139.44259.74, 16717206

[17] Tombaugh TN McIntyre NJ. The mini-mental state examination: a comprehensive review. J Am Geriatr Soc. (1992) 40:922–35. doi: 10.1111/j.1532-5415.1992.tb01992.x, 1512391

[18] Nasreddine ZS Phillips NA Bédirian V Charbonneau S Whitehead V Collin I . The Montreal Cognitive Assessment, MoCA: a brief screening tool for mild cognitive impairment. J Am Geriatr Soc. (2005) 53:695–9. doi: 10.1111/j.1532-5415.2005.53221.x, 15817019

[19] Hamilton M. The assessment of anxiety states by rating. Br J Med Psychol. (1959) 32:50–5. doi: 10.1111/j.2044-8341.1959.tb00467.x, 13638508

[20] Hamilton M. Development of a rating scale for primary depressive illness. Br J Soc Clin Psychol. (1967) 6:278–96. doi: 10.1111/j.2044-8260.1967.tb00530.x, 6080235

[21] Smith SM. Fast robust automated brain extraction. Hum Brain Mapp. (2002) 17:143–55. doi: 10.1002/hbm.10062, 12391568 PMC6871816

[22] Jenkinson M Beckmann CF Behrens TE Woolrich MW Smith SM. FSL. NeuroImage. (2012) 62:782–90. doi: 10.1016/j.neuroimage.2011.09.015 21979382

[23] Behrens TE Woolrich MW Jenkinson M Johansen-Berg H Nunes RG Clare S . Characterization and propagation of uncertainty in diffusion-weighted MR imaging. Magn Reson Med. (2003) 50:1077–88. doi: 10.1002/mrm.10609, 14587019

[24] Gagnon JF Postuma RB Joncas S Desjardins C Latreille V. The Montreal Cognitive Assessment: a screening tool for mild cognitive impairment in REM sleep behavior disorder. Mov Disord. (2010) 25:936–40. doi: 10.1002/mds.23079, 20310038

[25] Chun CT Seward K Patterson A Melton A MacDonald-Wicks L. Evaluation of available cognitive tools used to measure mild cognitive decline: a scoping review. Nutrients. (2021) 13:3974. doi: 10.3390/nu13113974, 34836228 PMC8623828

[26] Yang Z Abdul Rashid NA Quek YF Lam M See YM Maniam Y . Montreal Cognitive Assessment as a screening instrument for cognitive impairments in schizophrenia. Schizophr Res. (2018) 199:58–63. doi: 10.1016/j.schres.2018.03.008, 29549976

[27] Liew TM Feng L Gao Q Ng TP Yap P. Diagnostic utility of Montreal Cognitive Assessment in the fifth edition of diagnostic and statistical manual of mental disorders: major and mild neurocognitive disorders. J Am Med Dir Assoc. (2015) 16:144–8. doi: 10.1016/j.jamda.2014.07.021, 25282632

[28] Folstein MF Folstein SE McHugh PR. “Mini-mental state”. A practical method for grading the cognitive state of patients for the clinician. J Psychiatr Res. (1975) 12:189–98. doi: 10.1016/0022-3956(75)90026-6, 1202204

[29] Lu J Li D Li F Zhou A Wang F Zuo X . Montreal cognitive assessment in detecting cognitive impairment in Chinese elderly individuals: a population-based study. J Geriatr Psychiatry Neurol. (2011) 24:184–90. doi: 10.1177/0891988711422528, 22228824

[30] Su Y Dong J Sun J Zhang Y Ma S Li M . Cognitive function assessed by Mini-Mental State Examination and risk of all-cause mortality: a community-based prospective cohort study. BMC Geriatr. (2021) 21:524. doi: 10.1186/s12877-021-02471-9, 34600472 PMC8487495

[31] Philippot A Dubois V Lambrechts K Grogna D Robert A Jonckheer U . Impact of physical exercise on depression and anxiety in adolescent inpatients: a randomized controlled trial. J Affect Disord. (2022) 301:145–53. doi: 10.1016/j.jad.2022.01.011, 35007642

[32] Rodriguez-Seijas C Thompson JS Diehl JM Zimmerman M. A comparison of the dimensionality of the Hamilton Rating Scale for anxiety and the DSM-5 Anxious-Distress Specifier Interview. Psychiatry Res. (2020) 284:112788. doi: 10.1016/j.psychres.2020.112788, 31978629

[33] Matza LS Morlock R Sexton C Malley K Feltner D. Identifying HAM-A cutoffs for mild, moderate, and severe generalized anxiety disorder. Int J Methods Psychiatr Res. (2010) 19:223–32. doi: 10.1002/mpr.323, 20718076 PMC6878292

[34] Carrozzino D Patierno C Fava GA Guidi J. The Hamilton Rating Scales for depression: a critical review of clinimetric properties of different versions. Psychother Psychosom. (2020) 89:133–50. doi: 10.1159/000506879, 32289809

[35] Trajković G Starčević V Latas M Leštarević M Ille T Bukumirić Z . Reliability of the Hamilton Rating Scale for Depression: a meta-analysis over a period of 49 years. Psychiatry Res. (2011) 189:1–9. doi: 10.1016/j.psychres.2010.12.007, 21276619

[36] Van Overwalle F Ma Q Heleven E. The posterior Crus II cerebellum is specialized for social mentalizing and emotional self-experiences: a meta-analysis. Soc Cogn Affect Neurosci. (2020) 15:905–28. doi: 10.1093/scan/nsaa124, 32888303 PMC7851889

[37] Habas C Kamdar N Nguyen D Prater K Beckmann CF Menon V . Distinct cerebellar contributions to intrinsic connectivity networks. J Neurosci. (2009) 29:8586–94. doi: 10.1523/JNEUROSCI.1868-09.2009, 19571149 PMC2742620

[38] Bigaut K Achard S Hemmert C Baloglu S Kremer L Collongues N . Resting-state functional MRI demonstrates brain network reorganization in neuromyelitis optica spectrum disorder (NMOSD). PLoS One. (2019) 14:e0211465. doi: 10.1371/journal.pone.0211465, 30695058 PMC6351056

[39] Puledda F Bruchhage M O'Daly O Ffytche D Williams SCR Goadsby PJ. Occipital cortex and cerebellum gray matter changes in visual snow syndrome. Neurology. (2020) 95:e1792–9. doi: 10.1212/WNL.0000000000010530, 32759201 PMC7682819

[40] Cai H Zhu J Zhang N Wang Q Zhang C Yang C . Subregional structural and connectivity damage in the visual cortex in neuromyelitis optica. Sci Rep. (2017) 7:41914. doi: 10.1038/srep41914, 28157198 PMC5291226

[41] Chavarro VS Bellmann-Strobl J Zimmermann HG Scheel M Chien C Oertel FC . Visual system damage and network maladaptation are associated with cognitive performance in neuromyelitis optica spectrum disorders. Mult Scler Relat Disord. (2020) 45:102406. doi: 10.1016/j.msard.2020.102406, 32707533

[42] Wu Y Zhong L Geng J. Visual impairment in neuromyelitis optica spectrum disorders (NMOSD). J Chem Neuroanat. (2019) 97:66–70. doi: 10.1016/j.jchemneu.2019.01.012, 30716473

[43] Allen G McColl R Barnard H Ringe WK Fleckenstein J Cullum CM. Magnetic resonance imaging of cerebellar-prefrontal and cerebellar-parietal functional connectivity. NeuroImage. (2005) 28:39–48. doi: 10.1016/j.neuroimage.2005.06.013, 16023375

[44] O’Reilly JX Beckmann CF Tomassini V Ramnani N Johansen-Berg H. Distinct and overlapping functional zones in the cerebellum defined by resting state functional connectivity. Cereb Cortex. (2010) 20:953–65. doi: 10.1093/cercor/bhp157, 19684249 PMC2837094

[45] Caligiore D Pezzulo G Baldassarre G Bostan AC Strick PL Doya K . Consensus paper: towards a systems-level view of cerebellar function: the interplay between cerebellum, basal ganglia, and cortex. Cerebellum. (2017) 16:203–29. doi: 10.1007/s12311-016-0763-3, 26873754 PMC5243918

[46] Schmahmann JD. The cerebellum and cognition. Neurosci Lett. (2019) 688:62–75. doi: 10.1016/j.neulet.2018.07.005, 29997061

[47] Tang F Zhu D Ma W Yao Q Li Q Shi J. Differences changes in cerebellar functional connectivity between mild cognitive impairment and Alzheimer’s disease: a seed-based approach. Front Neurol. (2021) 12:645171. doi: 10.3389/fneur.2021.645171, 34220669 PMC8248670

[48] Zhang J Cortese R De Stefano N Giorgio A. Structural and functional connectivity substrates of cognitive impairment in multiple sclerosis. Front Neurol. (2021) 12:671894. doi: 10.3389/fneur.2021.671894, 34305785 PMC8297166

[49] Roberts G Lord A Frankland A Wright A Lau P Levy F . Functional dysconnection of the inferior frontal gyrus in young people with bipolar disorder or at genetic high risk. Biol Psychiatry. (2017) 81:718–27. doi: 10.1016/j.biopsych.2016.08.018, 28031150

[50] Liakakis G Nickel J Seitz RJ. Diversity of the inferior frontal gyrus—a meta-analysis of neuroimaging studies. Behav Brain Res. (2011) 225:341–7. doi: 10.1016/j.bbr.2011.06.022, 21729721

[51] Yang Y Rui Q Han S Wu X Wang X Wu P . Reduced GABA levels in the medial prefrontal cortex are associated with cognitive impairment in patients with NMOSD. Mult Scler Relat Disord. (2022) 58:103496. doi: 10.1016/j.msard.2022.103496, 35032882

[52] Pichiecchio A Tavazzi E Poloni G Ponzio M Palesi F Pasin M . Advanced magnetic resonance imaging of neuromyelitis optica: a multiparametric approach. Mult Scler. (2012) 18:817–24. doi: 10.1177/1352458511431072, 22183930

[53] Pierpaoli C Barnett A Pajevic S Chen R Penix LR Virta A . Water diffusion changes in Wallerian degeneration and their dependence on white matter architecture. NeuroImage. (2001) 13:1174–85. doi: 10.1006/nimg.2001.0765, 11352623

[54] Lennon VA Kryzer TJ Pittock SJ Verkman AS Hinson SR. IgG marker of optic-spinal multiple sclerosis binds to the aquaporin-4 water channel. J Exp Med. (2005) 202:473–7. doi: 10.1084/jem.20050304, 16087714 PMC2212860

[55] Li Y Wang T Zhang T Lin Z Li Y Guo R . Fast high-resolution metabolic imaging of acute stroke with 3D magnetic resonance spectroscopy. Brain. (2020) 143:3225–33. doi: 10.1093/brain/awaa264, 33141145 PMC7719019

[56] Moseley ME Kucharczyk J Mintorovitch J Cohen Y Kurhanewicz J Derugin N . Diffusion-weighted MR imaging of acute stroke: correlation with T2-weighted and magnetic susceptibility-enhanced MR imaging in cats. AJNR Am J Neuroradiol. (2024) 45:S8–S14. doi: 10.3174/ajnr.45-12.S8 2161612 PMC8367476

[57] Moseley ME Kucharczyk J Mintorovitch J Cohen Y Kurhanewicz J Derugin N . Diffusion-weighted MR imaging of acute stroke: correlation with T2-weighted and magnetic susceptibility-enhanced MR imaging in cats. AJNR Am J Neuroradiol. (1990) 11:423–9. 2161612 PMC8367476

[58] Deoni SC Dean DC 3rd O’Muircheartaigh J Dirks H Jerskey BA. Investigating white matter development in infancy and early childhood using myelin water faction and relaxation time mapping. NeuroImage. (2012) 63:1038–53. doi: 10.1016/j.neuroimage.2012.07.037 22884937 PMC3711836

[59] Sotiropoulos SN Jbabdi S Xu J Andersson JL Moeller S Auerbach EJ . Advances in diffusion MRI acquisition and processing in the human connectome project. NeuroImage. (2013) 80:125–43. doi: 10.1016/j.neuroimage.2013.05.057 23702418 PMC3720790

[60] Meng J Du J Diao X Zou Y. Effects of an evidence-based nursing intervention on prevention of anxiety and depression in the postpartum period. Stress Health. (2022) 38:435–42. doi: 10.1002/smi.3104, 34633141

