# Supplementary figures and images for: Cerebellar microstructural and functional connectivity changes in patients with neuromyelitis optica spectrum disorders and their correlation with cognitive function: a female-dominated multimodal MRI study
Source: Front Neurol. 2025 Dec 9;16:1647244. doi: 10.3389/fneur.2025.1647244 (PMC12722828; doi:10.3389/fneur.2025.1647244)

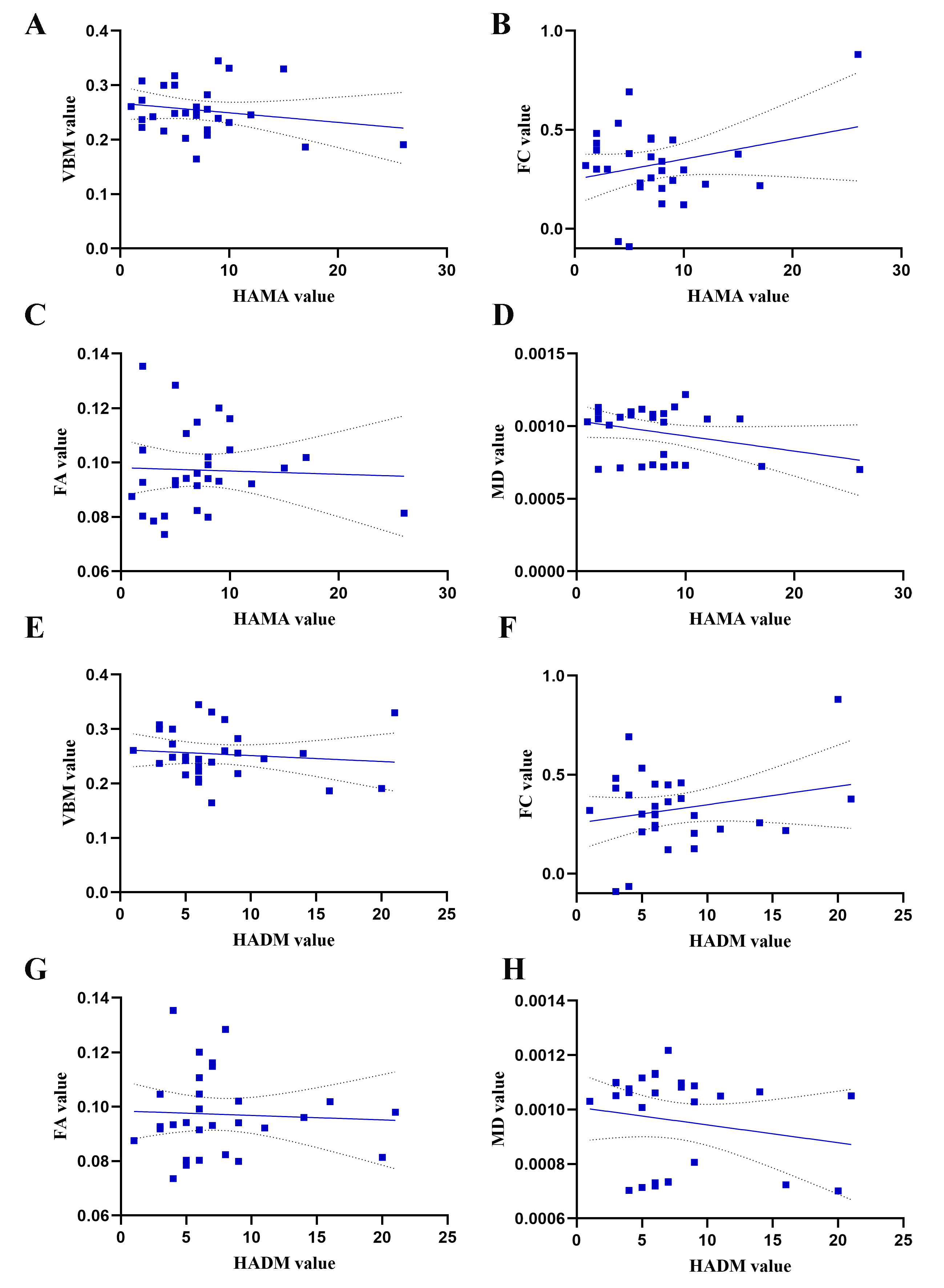

Supplement: Supplementary file 1 [file Image_1.tif]
